# Influence of Fetal-Type Posterior Cerebral Artery on Morphological Characteristics and Rupture Risk of Posterior Communicating Artery Aneurysms: A Radiomics Approach

**DOI:** 10.3390/jcm14113682

**Published:** 2025-05-24

**Authors:** Kunhee Han, Minu Nahm, Shin-Woong Ko, Hyeong-Joong Yi, Hyoung-Joon Chun, Young-Jun Lee, Sang Hyung Lee, Jaiyoung Ryu, Simon Song, Kyu-Sun Choi

**Affiliations:** 1 Department of Neurosurgery, Hanyang University Medical Center, Hanyang University College of Medicine, Seoul 04763, Republic of Korea; ns.khhan@gmail.com (K.H.);; 2 Center for Precision Medicine Platform Based on Smart Hemo-Dynamic Index, Seoul 04763, Republic of Korea; 3 Department of Radiology, Hanyang University Medical Center, Hanyang University College of Medicine, Seoul 04763, Republic of Korea; 4 Department of Neurosurgery, Jeju National University Hospital, Jeju National University College of Medicine, Jeju 63241, Republic of Korea; 5 Department of Mechanical Engineering, Korea University, Seoul 02841, Republic of Korea; 6 Department of Mechanical Convergence Engineering, Hanyang University, Seoul 04763, Republic of Korea

**Keywords:** fetal-type posterior cerebral artery, intracranial aneurysm, posterior communicating artery aneurysm, digital subtraction angiography, magnetic resonance angiography, radiomics, non-sphericity index

## Abstract

**Background/Objectives:** The fetal-type posterior cerebral artery (fetal PCA) is an anatomical variant that alters hemodynamics and may influence posterior communicating artery (PCoA) aneurysm rupture risk. Aneurysm shape and size irregularity are key rupture predictors. This study investigates the impact of fetal PCA on PCoA aneurysm morphology and rupture risk using a radiomics-based approach. **Methods:** We retrospectively analyzed 87 patients with PCoA aneurysms (39 ruptured, 48 unruptured) treated at a tertiary center (January 2017–December 2022). Seventeen morphological parameters and 18 radiomic features were extracted per aneurysm. Patients were grouped by fetal PCA presence. Logistic regression and receiver operating characteristic (ROC) analyses identified rupture predictors. **Results:** Of 87 aneurysms, 38 had fetal PCA (24 ruptured, 14 unruptured), and 49 did not (15 ruptured, 34 unruptured). Fetal PCA was significantly associated with rupture (odds ratio [OR]: 3.28, *p* = 0.018). A higher non-sphericity index (NSI) correlated with rupture risk (OR: 3.35, *p* = 0.016). In non-fetal PCA aneurysms, size-related parameters such as height (6.83 ± 3.54 vs. 4.88 ± 2.57 mm, *p* = 0.034) and area (190.84 ± 167.08 vs. 107.94 ± 103.10 mm^2^, *p* = 0.046) were key rupture predictors. In fetal PCA aneurysms, flow-related parameters like vessel angle (55.78 ± 31.39 vs. 38.51 ± 24.71, *p* = 0.035) were more influential. ROC analysis showed good discriminatory power, with an area under the curve: 0.726 for fetal PCA and 0.706 for NSI. **Conclusions:** Fetal PCA influences PCoA aneurysm rupture risk and morphology. NSI is a reliable rupture marker. Integrating morphological and anatomical data may improve rupture risk assessment and clinical decision-making.

## 1. Introduction

Posterior communicating artery (PCoA) aneurysms account for 15–25% of intracranial aneurysms and have a higher rupture risk than aneurysms in other locations [1,2]. Ruptured intracranial aneurysms (IAs) are a leading cause of non-traumatic subarachnoid hemorrhage (SAH), which has high morbidity and mortality [3]. Despite advances in diagnostic imaging, accurately predicting rupture risk remains a major clinical challenge. Various clinical, morphological, and hemodynamic factors have been identified as predictors of IA rupture [2,4,5,6].

The fetal-type posterior cerebral artery (fetal PCA) is a common anatomical variant in which the PCA is primarily supplied by the internal carotid artery rather than the vertebrobasilar system [7,8]. Present in 11–46% of the population, fetal PCA is associated with significant hemodynamic alterations [9,10,11]. These changes include increased blood flow and vessel wall stress, which contribute to PCoA aneurysm formation, growth, and rupture [8,11,12,13,14]. Studies have identified fetal PCA as an independent risk factor for PCoA aneurysm rupture, alongside morphological parameters such as aneurysm size and aspect ratio [8,12,13,14,15].

Radiomics is an emerging computer-assisted technique that objectively extracts high-throughput quantitative features—including shape, intensity, and texture—from biomedical images [16,17]. In parallel, radiomics—a quantitative image analysis approach—has emerged as a promising method to capture complex morphological characteristics beyond conventional measurements. In particular, shape irregularity has been increasingly recognized as a critical factor in rupture prediction. Its application in cerebrovascular disease has expanded in recent years, with multiple studies linking radiomic features to IA rupture risk [18,19]. Radiomics, when combined with artificial intelligence (AI), enables automated image analysis and risk prediction. Recent studies have demonstrated its growing role in cerebrovascular imaging, offering objective tools for rupture risk stratification [20]. However, few studies have integrated radiomics with morphological parameters to assess PCoA aneurysm rupture risk, particularly in the context of fetal PCA.

This study addresses this gap by investigating the influence of fetal PCA on the morphological characteristics and rupture risk of PCoA aneurysms using a radiomics-based approach. We hypothesize that integrating radiomics with morphological and anatomical data will enhance rupture risk prediction and improve clinical decision-making.

## 2. Materials and Methods

### 2.1. Ethics Approval and Patient Consent

This retrospective study and all the procedures within were conducted in accordance with the ethical standards of the institutional research committee and the Declaration of Helsinki and approved by the Institutional Review Board of the local institution (approval number: [HYUH2023-04-028]). The requirement for informed consent was waived owing to the retrospective nature of the research and the use of anonymized patient data.

### 2.2. Study Population and Clinical Characteristics

Between January 2017 and December 2022, 782 patients with ruptured or unruptured IAs were treated at a single tertiary center. Among them, 685 patients were excluded due to aneurysms located in sites other than the PCoA, resulting in 97 patients with PCoA aneurysms who underwent surgical or endovascular treatment. We excluded 10 patients due to the absence of preoperative digital subtraction angiography (DSA) or magnetic resonance angiography (MRA), yielding a final cohort of 87 patients. Baseline clinical characteristics included age, sex, body mass index (BMI), medical history (hypertension, diabetes mellitus [DM], and hyperlipidemia), and tobacco use. Hypertension was defined as systolic blood pressure ≥ 140 mmHg, diastolic blood pressure ≥ 90 mmHg, or antihypertensive medication use. Hyperlipidemia was defined as a total cholesterol level ≥ 240 mg/dL or the use of antihyperlipidemic medications.

### 2.3. Radiological Evaluation and Parameter Measurement

**Figure 1:**
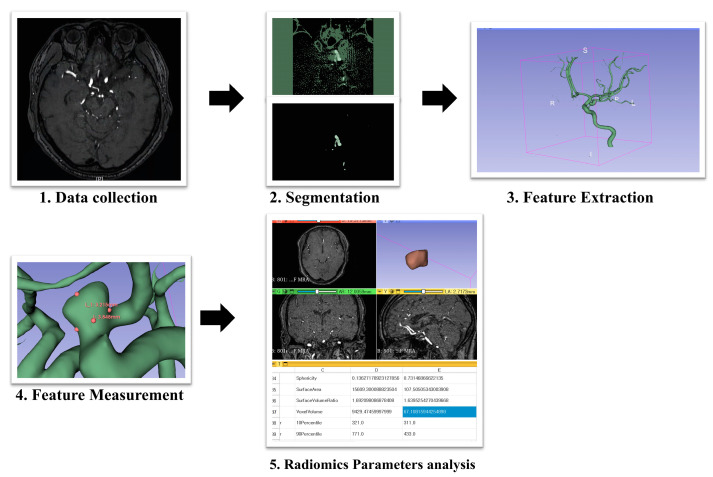
Overview of the study methodology.

All patients underwent DSA, including 3D rotational angiography or MRA, reviewed by a single neuroradiologist. Seventeen established morphological parameters, as defined in previous studies (Supplementary Table S1) [21,22], were measured from angiographic source images. To ensure measurement reliability, all parameters were independently measured by a trained neurosurgeon with over 5 years of experience and subsequently reviewed for consistency by a second neurosurgeon. Interobserver discrepancies were resolved through consensus. Prior to formal measurements, five randomly selected cases were used to calibrate inter-rater agreement, yielding an intraclass correlation coefficient (ICC) > 0.85 across all metrics. Aneurysm segmentation was performed using 3D Slicer software (version 5.6.2; https://slicer.org) by a single neurosurgeon. Radiomic features were extracted using the PyRadiomics module in Python (version 3.9; https://www.python.org/) [19,23]. Eighteen radiomics parameters were pre-selected based on prior studies reporting their association with aneurysm rupture risk (Supplementary Table S1) [18,19]. All features were shape-based and derived from 3D segmentation masks of the aneurysm. To ensure comparability across imaging datasets, all source images were resampled to an isotropic voxel size of 1 × 1 × 1 mm^3^ using linear interpolation prior to feature extraction. Intensity normalization was not performed, as no intensity- or texture-based features were included in this study. Radiomic feature extraction was conducted in compliance with the Image Biomarker Standardisation Initiative guidelines to enhance reproducibility. In addition, quality control procedures were adapted from Liu et al. [19] to exclude segmentations with artifacts, poor contrast, or biologically implausible shape outliers. The overall study workflow is summarized in Figure 1.

### 2.4. Statistical Analyses

Statistical analyses were performed using SPSS software (version 29; IBM, Armonk, NY, USA). The normality of continuous variables was assessed using the Shapiro–Wilk test. Variables following a normal distribution are presented as mean ± standard deviation (SD), while non-normally distributed variables are expressed as median and interquartile range (IQR). Categorical variables are reported as frequencies (percentages). For between-group comparisons, we used Student’s *t*-test or the Mann–Whitney U test for continuous variables, depending on data distribution, and the chi-squared test or Fisher’s exact test for categorical variables, depending on expected cell counts. All tests were two-sided, and a *p*-value < 0.05 was considered statistically significant. To identify potential predictors of aneurysm rupture, univariate logistic regression analyses were first performed. Variables with a *p*-value < 0.10 in the univariate analysis were then included in a multivariate logistic regression model using a stepwise forward selection method. Odds ratios (ORs) with 95% confidence intervals (CIs) were calculated for each variable.

To evaluate model discrimination, receiver operating characteristic (ROC) curve analysis was conducted, and the area under the curve (AUC) was computed. To reduce overfitting and improve generalizability, we performed 10-fold cross-validation during the multivariate analysis. This validation approach enhanced the robustness and reproducibility of the predictive model, particularly given the relatively small sample size.

## 3. Results

### 3.1. Baseline Characteristics and Univariate Analysis of Ruptured Status

Table 1 summarizes the baseline characteristics of the 87 patients with PCoA aneurysms included in this study. Among them, 39 (44.8%) had ruptured aneurysms, while 48 (55.2%) were unruptured. The mean patient age was 64.43 ± 11.4 years (range 27–91), and 13 (14.9%) were male. Twenty-nine (33.3%) were current smokers. The prevalence of hypertension, DM, and hyperlipidemia was 55 (63.2%), 14 (16.1%), and 26 (29.9%), respectively. There were no significant differences between the ruptured and unruptured groups in terms of sex (15.4% vs. 14.6%, *p* = 0.917), age (63.18 ± 11.77 vs. 65.56 ± 11.14 years, *p* = 0.168), or BMI (22.44 ± 3.05 vs. 23.16 ± 3.74, *p* = 0.159). Among comorbidities, DM was significantly less common in the ruptured group (5.1% vs. 25.0%, *p* = 0.012). However, hypertension (59.0% vs. 33.3%, *p* = 0.459), hyperlipidemia (25.6% vs. 33.3%, *p* = 0.436), and smoking status (23.1% vs. 16.7%, *p* = 0.413) were not significantly associated with the rupture status.

### 3.2. Univariate Analysis of Morphological Parameters

Table 2 summarizes the univariate statistical analysis of the established morphological parameters distinguishing ruptured from unruptured aneurysms. Ruptured aneurysms were significantly larger across multiple dimensions: height (6.62 ± 3.37 vs. 5.24 ± 2.62 mm, *p* = 0.020), width (5.86 ± 2.98 vs. 4.71 ± 2.22 mm, *p* = 0.024), maximum size (8.13 ± 3.96 vs. 6.73 ± 3.11 mm, *p* = 0.035), area (171.58 ± 151.47 vs. 114.16 ± 100.43 mm^2^, *p* = 0.023), and volume (191.58 ± 249.19 vs. 103.41 ± 130.92 mm^3^, *p* = 0.025). These findings suggest that larger aneurysms, in terms of height, width, area, and volume, are more likely to rupture.

### 3.3. Univariate Analysis of Radiomics Factors

Table 3 presents the univariate statistical analysis of radiomics factors differentiating ruptured from unruptured aneurysms. Ruptured aneurysms exhibited significantly larger dimensions across multiple measurements: major axis length (7.67 ± 3.19 mm vs. 6.17 ± 2.84 mm, *p* = 0.022), maximum 2D diameter column (7.63 ± 3.43 mm vs. 6.5 ± 2.64 mm, *p* = 0.026), maximum 2D diameter row (8.18 ± 3.99 mm vs. 6.42 ± 3.01 mm, *p* = 0.012), maximum 2D diameter slice (7.83 ± 3.52 mm vs. 6.34 ± 2.89 mm, *p* = 0.013), maximum 3D diameter (10.3 ± 5.54 mm vs. 7.34 ± 3.11 mm, *p* = 0.047), and minor axis length (5.35 ± 2.43 mm vs. 4.44 ± 1.81 mm, *p* = 0.01). Shape irregularity parameters also significantly differed between the two groups, with ruptured aneurysms demonstrating greater surface complexity: surface volume ratio (1.62 ± 0.91 vs. 1.76 ± 0.69, *p* = 0.018), surface area (171.66 ± 151.4 mm^2^ vs. 114.57 ± 100.11 mm^2^, *p* = 0.016), sphericity (0.77 ± 0.05 vs. 0.8 ± 0.05, *p* = 0.013), non-sphericity index (NSI; 0.37 ± 0.04 vs. 0.33 ± 0.07, *p* = 0.003), and undulation index (0.08 ± 0.07 vs. 0.05 ± 0.07, *p* = 0.031). However, parameters such as elongation and flatness were not significantly associated with rupture status. These results suggest that larger aneurysm size and increased geometric irregularity are strong indicators of rupture risk.

### 3.4. Association Between Fetal PCA and Aneurysm Rupture

Fetal PCA was identified in 38 aneurysms (43.7%), with a significantly higher prevalence in the ruptured (24 ruptured, 61.5% vs. 14 unruptured, 29.2%, *p* < 0.001). Univariate analysis confirmed that the presence of fetal PCA was significantly associated with aneurysm rupture.

### 3.5. Differences Based on Fetal PCA Status

Table 4 and Table 5 present the univariate analysis of established morphological and radiomics parameters in ruptured and unruptured aneurysms stratified by fetal PCA status.

In aneurysms without fetal PCA, ruptured aneurysms exhibited significantly larger size-related parameters: height (6.83 ± 3.54 vs. 4.88 ± 2.57 mm, *p* = 0.034), surface area (190.84 ± 167.08 vs. 107.94 ± 103.10 mm^2^, *p* = 0.046), and aspect ratio (1.68 ± 0.8 vs. 1.22 ± 0.49, *p* = 0.027), with borderline significance for volume (223.19 ± 272.07 vs. 95.91 ± 126.53 mm^3^, *p* = 0.051). Radiomics analysis further highlighted a significantly larger maximum 2D diameter (slice) in ruptured aneurysms (7.84 ± 3.67 vs. 5.97 ± 2.86 mm, *p* = 0.047).

These results suggest that in aneurysms without fetal PCA, size-related parameters play a more dominant role in predicting rupture risk.

**Figure 2:**
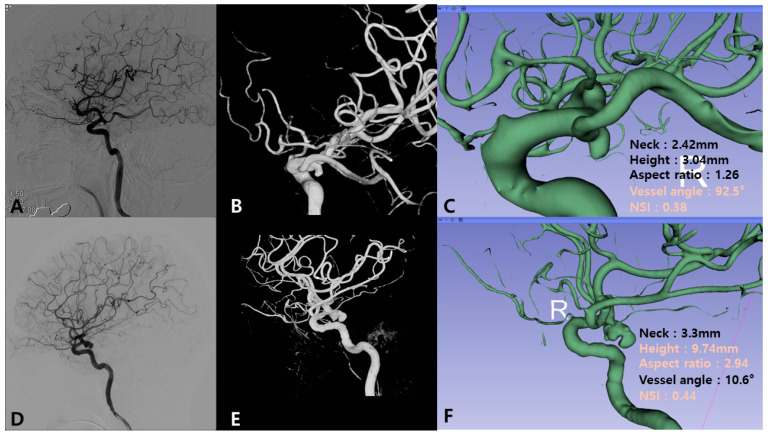
Illustrative cases of ruptured PCoA aneurysms with and without fetal PCA. (**A**–**C**) Ruptured aneurysm with fetal PCA. (**A**) Lateral view of the digital subtraction angiography (DSA) showing the ruptured posterior communicating artery (PCoA) aneurysm with fetal posterior cerebral artery (PCA). (**B**) Three-dimensional rotational angiography (3DRA) showing a small and irregularly shaped aneurysm. (**C**) The 3D Slicer analysis shows a high vessel angle (92.5°) and non-sphericity index (NSI; 0.38). (**D**–**F**) Ruptured aneurysm without fetal PCA. (**D**) Lateral view of the DSA showing the ruptured PCoA aneurysm with non-fetal PCA. (**E**) 3DRA shows a large and irregularly shaped aneurysm. (**F**) The 3D Slicer analysis showing large-sized-related parameters (height 9.74 mm and aspect ratio 2.94) and NSI (0.44).

In contrast, among aneurysms with fetal PCA, ruptured aneurysms exhibited different morphological characteristics, particularly those influencing hemodynamics: vessel angle (55.78 ± 31.39 vs. 38.51 ± 24.71°, *p* = 0.035) and maximum 3D diameter (10.96 ± 16.14 vs. 7.75 ± 2.81 mm, *p* = 0.037). These findings indicate that in the presence of fetal PCA, geometric factors influencing flow dynamics, such as vessel angle rather than size alone, may play a more critical role in rupture risk. Regardless of fetal PCA status, the NSI remained a significant predictor of rupture: without fetal PCA: *p* = 0.011; with fetal PCA: *p* = 0.019. This reinforces the importance of aneurysm shape irregularity as a key determinant of rupture risk (Figure 2).

### 3.6. Multivariate Logistic Regression Analysis

**Figure 3:**
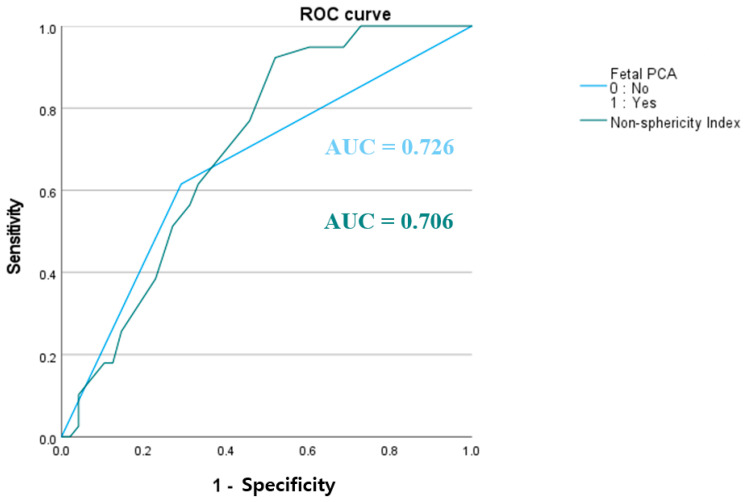
Receiver operating characteristic (ROC) curve for predicting aneurysm rupture. The ROC curves illustrate the predictive performance of fetal-type posterior cerebral artery (PCA; blue line, area under the curve [AUC] = 0.726) and the non-sphericity index (green line, AUC = 0.706) for distinguishing ruptured from unruptured aneurysms. The x-axis represents 1—specificity, and the y-axis represents sensitivity. Both variables demonstrated discriminatory ability for aneurysm rupture.

Multivariate logistic regression identified the NSI (OR: 3.954, *p* = 0.002) and fetal PCA (OR: 3.85, *p* = 0.005) as significant predictors of aneurysm rupture risk. ROC curve analysis demonstrated that the area under the curve (AUC) values for the NSI and fetal PCA were 0.706 and 0.726, respectively, indicating their moderate discriminatory ability to distinguish ruptured from unruptured aneurysms (Figure 3). To facilitate interpretation, the raw NSI values (0–1) were scaled by a factor of 10, yielding values between 2.2 and 5. According to the logistic regression model, each 0.1 increase in the NSI corresponded to a 3.954-fold increase in the odds of rupture. Furthermore, having a fetal PCA rather than a non-fetal PCA increased the odds of rupture by approximately 3.85. Overall, these findings suggest that both aneurysm shape irregularity (NSI) and fetal PCA play crucial roles in rupture risk for PCoA aneurysms.

## 4. Discussion

In this study, we integrated conventional morphological parameters and radiomics analysis to assess the rupture risk of PCoA aneurysms, with a particular focus on the presence of fetal PCA. Our findings highlight the significance of fetal PCA and NSI as key predictors of rupture risk, offering insights for risk stratification and clinical decision-making.

### 4.1. Impact of Fetal PCA on Aneurysm Rupture Risk

IAs are classified into bifurcation-type or sidewall-type aneurysms [24,25]. Sidewall aneurysms arise from a single parent vessel or the origin of a significantly smaller branch, while bifurcation aneurysms form at major bifurcations or involve a branch vessel larger than the parent vessel [11,24,25]. Based on this classification, PCoA aneurysms with fetal PCA are categorized as bifurcation type, while those without fetal PCA are sidewall type. Baharoglu et al. [24] reported that bifurcation aneurysms accounted for 66.3% of all ruptured aneurysms, emphasizing their distinct morphological and hemodynamic characteristics compared to sidewall aneurysms. Based on these findings, we hypothesized that PCoA aneurysms originating from fetal PCA (bifurcation type) may have a higher rupture risk than those originating from non-fetal PCA (sidewall type). Our findings confirmed this association. Fetal PCA was significantly linked to an increased risk of PCoA aneurysm rupture. This aligns with previous studies suggesting that fetal PCA can alter hemodynamic flow patterns, which may lead to increased wall shear stress and greater vulnerability to aneurysm formation and rupture [11,13]. In this study, multivariate logistic regression further supported this relationship. A significantly higher proportion of fetal PCA was observed in the ruptured aneurysm group (OR: 3.28, 95% CI: 1.21–8.86, *p* = 0.018). These results suggest that fetal PCA may elevate rupture risk by inducing hemodynamic stress and compromising vessel wall stability. This highlights the importance of fetal PCA in risk stratification for PCoA aneurysms.

### 4.2. Differential Influence of Parameters on Rupture Risk Based on Fetal PCA Status

Our analysis revealed distinct predictors of rupture risk based on fetal PCA status. In aneurysms without fetal PCA, morphological parameters such as height, area, and volume were significant predictors of rupture, highlighting the role of aneurysms. Conversely, in aneurysms with fetal PCA, flow-related factors, particularly vessel angle, demonstrated a stronger association with rupture risk. One possible explanation is that fetal PCA alters the local hemodynamic environment, leading to higher-velocity jets or turbulence near the aneurysm neck. This shift in flow dynamics may increase wall shear stress and vessel wall instability, making geometric factors more critical than absolute aneurysm size [11,13,26]. Even minor variations in vessel angle could significantly elevate hemodynamic stress, thereby increasing rupture risk. These findings suggest that while size-based parameters remain valuable for predicting rupture in non-fetal PCA aneurysms, flow-related parameters should be prioritized in fetal PCA aneurysms. This underscores the need for tailored risk assessment and treatment strategies based on fetal PCA status. Although further validation is warranted, these observations may cautiously support closer monitoring or individualized treatment planning in patients with fetal PCA-associated PCoA aneurysms, even when the aneurysm appears relatively small.

### 4.3. Utility of Geometric Radiomics Parameters

Shape irregularity has been identified as a key predictor of aneurysm rupture risk [27,28,29]. Recent advances in radiomics have further enhanced rupture risk assessment by quantifying aneurysm shape characteristics. Zhang et al. [23] identified radiomics elongation as a predictor of angiographic occlusion status following flow diverter embolization for cerebral aneurysms. Liu et al. [19] found flatness to be the most significant predictor of aneurysm stability, achieving an AUC of 0.73. In our study, we identified the NSI as a significant radiomics-based morphological predictor of aneurysm rupture. The NSI is a dimensionless metric that quantifies deviation from an ideal spherical shape, with higher values indicating more irregular and complex surface geometry. Unlike traditional morphological descriptors that emphasize size or linear dimensions, the NSI captures the spatial heterogeneity of the aneurysm surface, which may reflect underlying biomechanical stress and structural instability. In our analysis, ruptured aneurysms exhibited significantly higher NSI values, and multivariate analysis confirmed the NSI as an independent predictor of rupture (OR: 3.35, 95% CI: 1.21–8.85, *p* = 0.016). These findings align with prior research emphasizing the role of aneurysm shape in rupture risk assessment. Benemerito et al. [29] also identified the NSI and maximum oscillatory shear index as strong rupture predictors, reinforcing the need to integrate shape analysis with hemodynamic factors in rupture risk stratification. In summary, the NSI serves as a valuable tool for rupture risk assessment and clinical decision-making. Future studies combining the NSI with hemodynamic parameters, such as wall shear stress, may further refine rupture risk prediction models, improving patient management strategies.

### 4.4. Limitations

Our study has some limitations. First, multicenter, prospective studies with larger cohorts are needed to validate our results and improve generalizability. Second, the relatively small sample size may have limited the statistical power of our analysis. Third, variations in imaging acquisition protocols may have influenced the reproducibility of certain radiomic features. Standardized imaging and segmentation protocols should be implemented in future studies to ensure consistency and comparability. Fourth, although the model was internally validated using 10-fold cross-validation, external validation with independent datasets was not performed, which may limit generalizability. Finally, although this study focused on fetal PCA and morphological parameters, it did not directly assess hemodynamic factors such as wall shear stress and flow dynamics. Since hemodynamic stress plays a critical role in aneurysm rupture, integrating computational fluid dynamics analyses with radiomics and anatomical data could provide deeper insights into the underlying mechanisms and improve risk stratification. Future research should explore multimodal models that combine morphological radiomic features with hemodynamic metrics to enhance predictive performance. Alternatively, increased flow demand from an enlarged posterior territory in fetal PCA may underlie the observed morphological and rupture differences. Further hemodynamic studies are warranted.

## 5. Conclusions

This study identified the presence of fetal PCA and higher NSI values as significant predictors of PCoA aneurysm rupture risk. In fetal PCA-associated PCoA aneurysms, flow-related factors appear to play a more critical role in rupture prediction, whereas size-based parameters remain more relevant for aneurysms without fetal PCA. The NSI, which quantifies shape irregularity, differed significantly between ruptured and unruptured aneurysms, reinforcing its importance in rupture risk stratification. Our findings suggest that integrating radiomics-based shape assessments with anatomical features could enhance rupture risk evaluations. Larger prospective studies are needed to validate these findings and further explore the interplay between morphological and hemodynamic factors in PCoA aneurysm rupture.

## Figures and Tables

**Table 1:** Baseline characteristics of patients.

Parameters	Ruptured (*n* = 39)	Unruptured (*n* = 48)	*p*-Value	AUC	Univariate Analysis
Sex (Male)	6 (15.4%)	7 (14.6%)	0.917	0.496	0.917
Age (years, mean ± SD)	63.18 ± 11.77	65.56 ± 11.1	0.168	0.436	0.283
BMI (kg/m^2^)	22.44 ± 3.05	23.16 ± 3.74	0.159	0.458	0.546
Hypertension	23 (59.0%)	32 (66.7%)	0.459	0.462	0.459
Diabetes mellitus	2 (5.1%)	12 (25.0%)	0.012	0.401	0.012
Hyperlipidemia	10 (25.6%)	16 (33.3%)	0.436	0.462	0.436
Smoking	9 (23.1%)	8 (16.7%)	0.413	0.534	0.413

Abbreviations: SD, standard deviation; BMI, body mass index; AUC, area under the curve.

**Table 2:** Univariate statistical analysis of established morphological parameters for differentiating rupture status.

Parameters	Ruptured (*n* = 39)	Unruptured (*n* = 48)	*p*-Value	Univariate Analysis
Neck (mm)	4.47 ± 1.89	4.09 ± 1.39	0.156	0.450
Height (mm)	6.62 ± 3.37	5.24 ± 2.62	0.020	0.480
Width (mm)	5.86 ± 2.98	4.71 ± 2.22	0.024	0.480
Size maximum (mm)	8.13 ± 3.96	6.7 ± 3.11	0.035	0.419
Area (mm^2^)	171.58 ± 151.47	114.16 ± 100.43	0.023	0.450
Volume (mm^3^)	191.58 ± 249.19	103.41 ± 130.92	0.025	0.450
Height/Width	1.14 ± 0.21	1.12 ± 0.28	0.371	0.537
Flow angle (°)	116.94 ± 25.38	107.77 ± 28.3	0.058	0.390
Inclination angle (°)	97.78 ± 24.93	98.9 ± 18.97	0.409	0.513
Patent artery angle (°)	111.98 ± 26.7	111.33 ± 25.42	0.454	0.513
Vessel angle (°)	50.42 ± 33.39	37.94 ± 29.45	0.036	0.481
Proximal diameter of parent artery (mm)	4.65 ± 0.92	4.68 ± 0.72	0.446	0.390
Distal diameter of parent artery (mm)	3.47 ± 0.74	3.47 ± 0.71	0.494	0.419
Aspect ratio	1.52 ± 0.65	1.28 ± 0.53	0.036	0.527
Size ratio	2.86 ± 6.12	1.59 ± 2.92	0.119	0.633
Bottleneck factor	1.34 ± 0.53	1.15 ± 0.4	0.034	0.303
Fetal PCA	29 (74.4%)	14 (29.2%)	<0.001	<0.001

Abbreviations: AUC, area under the curve; PCA, posterior cerebral artery.

**Table 3:** Univariate statistical analysis of radiomics parameters for differentiating rupture status.

Parameters	Ruptured (*n* = 39)	Unruptured (*n* = 48)	*p*-Value	Univariate Analysis
Convex Hull Volume (Vch) (mm^3^)	213.11 ± 274.69	116.57 ± 157.31	0.028	0.450
Convex Hull Surface Area (Sch) (mm^2^)	157.1 ± 142.41	106.5 ± 96.23	0.031	0.450
Non-sphericity Index	0.37 ± 0.4	0.33 ± 0.7	0.003	0.216
Ellipticity Index	0.26 ± 0.02	0.26 ± 0.03	0.462	0.205
Undulation Index	0.08 ± 0.07	0.05 ± 0.07	0.031	0.253
Elongation	0.72 ± 0.15	0.75 ± 0.14	0.128	0.334
Flatness	0.6 ± 0.14	0.62 ± 0.13	0.227	0.655
LeastAxisLength (mm)	4.54 ± 2.22	3.7 ± 1.59	0.228	0.450
MajorAxisLength (mm)	7.67 ± 3.19	6.17 ± 2.84	0.022	0.450
Maximum2DDiameterColumn (mm)	7.6 ± 3.43	6.5 ± 2.64	0.026	0.439
Maximum2DDiameterRow (mm)	8.18 ± 3.99	6.42 ± 3.01	0.012	0.461
Maximum2DDiameterSlice (mm)	7.83 ± 3.52	6.34 ± 2.89	0.013	0.525
Maximum3DDiameter (mm)	10.3 ± 5.54	7.34 ± 3.11	0.047	0.480
MeshVolume	191.84 ± 249	103.47 ± 130.89	0.052	0.450
MinorAxisLength (mm)	5.35 ± 2.43	4.44 ± 1.81	0.010	0.450
Sphericity	0.77 ± 0.05	0.8 ± 0.05	0.013	0.234
Surfacearea1 (mm^2^)	171.66 ± 151.4	114.57 ± 100.11	0.016	0.450
SurfaceVolumeRatio	1.62 ± 0.91	1.76 ± 0.69	0.018	0.388

Abbreviations: AUC, area under the curve; PCA, posterior cerebral artery.

**Table 4:** Univariate statistical analysis of established morphological parameters for differentiating rupture status of fetal PCA.

	Non-Fetal PCA (*n* = 49)			Fetal PCA (*n* = 38)		
Parameters	Ruptured (*n* = 15)	Unruptured (*n* = 34)	*p*-Value	Ruptured (*n* = 24)	Unruptured (*n* = 14)	*p*-Value
Neck (mm)	4.22 ± 1.92	4.01 ± 1.58	0.356	4.62 ± 1.9	4.31 ± 0.8	0.243
Height (mm)	6.83 ± 3.54	4.88 ± 2.57	0.034	6.48 ± 3.33	6.11 ± 2.63	0.355
Width (mm)	6.03 ± 3.2	4.62 ± 2.39	0.070	5.76 ± 2.9	4.93 ± 1.8	0.142
Size maximum (mm)	8.48 ± 4.29	6.47 ± 3.24	0.059	7.91 ± 3.82	7.25 ± 2.78	0.274
Area (mm^2^)	190.84 ± 167.08	107.94 ± 103.1	0.046	159.55 ± 143.25	129.26 ± 95.59	0.220
Volume (mm^3^)	223.19 ± 272.07	95.91 ± 126.53	0.051	171.82 ± 237.64	121.65 ± 144.31	0.212
Height/Width	1.14 ± 0.18	1.08 ± 0.28	0.175	1.14 ± 0.23	1.23 ± 0.27	0.146
Flow angle (°)	116.97 ± 23.27	104.7 ± 29.66	0.064	116.93 ± 27.11	115.24 ± 24.04	0.422
Inclination angle (°)	100.27 ± 22.46	96.53 ± 18.03	0.288	96.22 ± 26.71	104.66 ± 20.65	0.142
Patent artery angle (°)	122.7 ± 21.13	113.06 ± 23.83	0.084	105.28 ± 28	107.14 ± 29.45	0.425
Vessel angle (°)	41.83 ± 35.75	37.7 ± 31.54	0.351	55.78 ± 31.39	38.51 ± 24.71	0.035
Proximal diameter of parent artery (mm)	4.4 ± 0.85	4.58 ± 0.67	0.234	4.81 ± 0.95	4.9 ± 0.8	0.374
Distal diameter of parent artery (mm)	3.49 ± 0.7	3.52 ± 0.68	0.457	3.45 ± 0.78	3.34 ± 0.81	0.344
Aspect ratio	1.68 ± 0.8	1.22 ± 0.49	0.027	1.42 ± 0.53	1.43 ± 0.62	0.475
Size ratio	2.63 ± 5.06	1.85 ± 3.43	0.295	3.01 ± 6.8	0.96 ± 0.64	0.078
Bottleneck factor	1.46 ± 0.61	1.14 ± 0.39	0.040	1.26 ± 0.47	1.16 ± 0.43	0.255

Abbreviations: PCA, posterior cerebral artery.

**Table 5:** Univariate statistical analysis of radiomics parameters for differentiating rupture status of fetal PCA.

	Non-Fetal PCA (*n* = 49)			Fetal PCA (*n* = 38)		
Parameters	Ruptured (*n* = 15)	Unruptured (*n* = 34)	*p*-Value	Ruptured (*n* = 24)	Unruptured (*n* = 14)	*p*-Value
Convex Hull Volume (Vch) (mm^3^)	253.89 ± 307.17	107.85 ± 148.58	0.049	187.62 ± 255.87	137.75 ± 180.96	0.244
Convex Hull Surface Area (Sch) (mm^2^)	176.5 ± 159.17	99.48 ± 94.74	0.049	144.97 ± 132.99	123.55 ± 101.26	0.290
Non-sphericity Index	3.8 ± 0.5	3.3 ± 0.7	0.003	03.7 ± 0.4	3.3 ± 0.6	0.019
Ellipticity Index	0.26 ± 0.02	0.26 ± 0.03	0.482	0.26 ± 0.02	0.26 ± 0.02	0.443
Undulation Index	0.11 ± 0.09	0.05 ± 0.07	0.011	0.06 ± 0.05	0.06 ± 0.06	0.491
Elongation	0.72 ± 0.13	0.75 ± 0.15	0.236	0.71 ± 0.16	0.76 ± 0.14	0.174
Flatness	0.61 ± 0.12	0.62 ± 0.14	0.383	0.6 ± 0.15	0.64 ± 0.1	0.193
LeastAxisLength (mm)	4.73 ± 2.56	3.53 ± 1.64	0.056	4.43 ± 2.03	4.13 ± 1.45	0.297
MajorAxisLength (mm)	7.7 ± 3.45	5.97 ± 2.94	0.053	7.65 ± 3.08	6.66 ± 2.61	0.151
Maximum2DDiameterColumn (mm)	7.7 ± 3.8	6.26 ± 2.75	0.101	7.54 ± 3.26	7.07 ± 2.35	0.305
Maximum2DDiameterRow (mm)	8.2 ± 4.05	6.3 ± 3.13	0.060	8.17 ± 4.04	6.7 ± 2.78	0.096
Maximum2DDiameterSlice (mm)	7.84 ± 3.67	5.97 ± 2.86	0.047	7.82 ± 3.5	7.24 ± 2.85	0.289
Maximum3DDiameter (mm)	9.26 ± 4.41	7.18 ± 3.25	0.058	10.96 ± 6.14	7.75 ± 2.81	0.037
MeshVolume (mm^3^)	223.27 ± 272.01	95.98 ± 126.49	0.051	172.2 ± 237.39	121.66 ± 144.31	0.210
MinorAxisLength (mm)	5.46 ± 2.71	4.27 ± 1.9	0.069	5.28 ± 2.29	4.87 ± 1.53	0.258
Sphericity	0.77 ± 0.06	0.8 ± 0.06	0.077	0.78 ± 0.04	0.81 ± 0.04	0.025
Surface Area (mm^2^)	190.84 ± 167.08	108.53 ± 102.68	0.047	159.67 ± 143.14	129.26 ± 95.59	0.219
SurfaceVolumeRatio	1.69 ± 1.16	1.86 ± 0.71	0.296	1.57 ± 0.73	1.51 ± 0.56	0.383

Abbreviations: PCA, posterior cerebral artery.

## Data Availability

The PyRadiomics scripts used for feature extraction are available upon request. Due to patient confidentiality and institutional regulations, imaging data cannot be shared publicly but may be made available in de-identified form upon reasonable request and with appropriate ethical approval.

## Supplementary Materials

The following supporting information can be downloaded at: https://www.mdpi.com/article/10.3390/jcm14113682/s1, Table S1: Definition of the parameters; Table S2: Multivariate Logistic Regression Analysis for Aneurysm Rupture Prediction.

## Abbreviations

The following abbreviations are used in this manuscript:

PCoA	posterior communicating artery
PCA	posterior cerebral artery
DSA	digital subtraction angiography
MRA	magnetic resonance angiography
NSI	non-sphericity index
ROC	receiver operating characteristic
IAs	intracranial aneurysms
DM	diabetes mellitus
AUC	area under the curve
