# Supplementary material for: Influence of Fetal-Type Posterior Cerebral Artery on Morphological Characteristics and Rupture Risk of Posterior Communicating Artery Aneurysms: A Radiomics Approach
Source: J Clin Med. 2025 May 24;14(11):3682. doi: 10.3390/jcm14113682 (PMC12155670; doi:10.3390/jcm14113682)
Supplement: Supplementary file 1 [file jcm-14-03682-s001.zip › jcm-3573639-supplementary.pdf]

Supplemental Digital Content

Supplemental Digital Content 1, Table S1. Definition of the parameters

**Established morphological parameters**	**Definition**
Size Maximum	Longest distance from the aneurysm neck plane to the dome
area	the surface area of the aneurysm sac
Volume	The 3D space enclosed by the aneurysm sac
Height/Width	Ratio of Height Maximum to Width (maximum width parallel to neck plane)
Flow angle	The angle between the inlet vessel centerline and the maximum length of the aneurysm
Inclination angle	The angle of inclination between the aneurysm and its neck plane
Patent artery angle	The angle between two parent arteries
Vessel angle	The angle between the inlet vessel centerline and the neck plane
Proximal diameter of parent artery	The diameter of the proximal parent vessel perpendicular to the flow, measured at a distance 1.5 times the parent vessel's diameter away from the aneurysm neck
Distal diameter of parent artery	The diameter of the distal parent vessel perpendicular to the flow, measured at a distance 1.5 times the parent vessel's diameter away from the aneurysm neck
Aspect Ratio	Ratio of Height Maximum to Neck
Size Ratio	Ratio of Height Maximum to Parent vessel size
BottleneckFactor	Ratio of WidthMaximum to Neck

**Radiomics parameters**	**Definition**
Convex Hull Volume (Vch)	The volume of the smallest convex shape that completely encloses the aneurysm sac
Convex Hull Surface Area (Sch)	The surface area of the smallest convex shape that completely encloses the aneurysm sac
Non-sphericity Index	A dimensionless metric that quantifies the deviation of the aneurysm shape from a perfect sphere, calculated as the ratio of the surface area of the aneurysm to the surface area of a sphere with the same volume

Ellipticity Index	A measure of the elongation of the aneurysm, calculated as the ratio of the longest axis to the shortest axis of the aneurysm
Undulation Index	A measure of surface irregularity, defined as the ratio of the aneurysm surface area to the convex hull surface area
Elongation	A measure shows relationship between the 2 largest principal components in the shape
Flatness	A measure shows the relationship between the largest and smallest principal components of the shape
LeastAxisLength	The shortest dimension of the aneurysm measured along the principal axes of its geometry
MajorAxisLength	The longest dimension of the aneurysm measured along the principal axes of its geometry
Maximum2DDiameterColumn	The largest pairwise Euclidean distance between surface mesh vertices in coronal plane
Maximum2DDiameterRow	The largest pairwise Euclidean distance between surface mesh vertices in sagittal plane
Maximum2DDiameterSlice	The largest pairwise Euclidean distance between surface mesh vertices in axial plane
Maximum3DDiameter	The largest pairwise Euclidean distance between surface mesh vertices
MeshVolume	The total volume of the shape
MinorAxisLength	The intermediate dimension of the aneurysm measured along the principal axes of its geometry
Sphericity	A measure of the roundness of the shape relative to a sphere
Surfacearea1	The total area of the shape
SurfaceVolumeRatio	The ratio of surface area to volume of a shape

3D, three dimensional

Supplemental Digital Content 2, Table S2. Multivariate Logistic Regression Analysis for Aneurysm Rupture Prediction

Variable	Adjusted OR	95% Confidence Interval	*P*-value
Height	1.21	0.98 – 1.50	0.071
Area	1.18	0.99 – 1.42	0.068
Volume	1.20	0.96 – 1.51	0.088

Vessel angle	1.27	0.96 – 1.60	0.061
Aspect ratio	1.31	0.99 – 1.69	0.058
Fetal PCA	3.85	1.48 – 9.98	0.005
Non-sphericity Index (NSI)	3.95	1.65 – 9.42	0.002
Undulation Index	1.15	0.91 – 1.44	0.102
Maximum3DDiameter	1.12	0.94 – 1.34	0.106
Surfacearea1	1.19	0.97 – 1.45	0.068
